# Mushroom β-Glucan Recovered from Antler-Type Fruiting Body of *Ganoderma lucidum* by Enzymatic Process and Its Potential Biological Activities for Cosmeceutical Applications

**DOI:** 10.3390/polym14194202

**Published:** 2022-10-07

**Authors:** Pilanee Vaithanomsat, Nutthamon Boonlum, Wantida Chaiyana, Singkome Tima, Songyot Anuchapreeda, Chanaporn Trakunjae, Waraporn Apiwatanapiwat, Phornphimon Janchai, Antika Boondaeng, Hataitip Nimitkeatkai, Amnat Jarerat

**Affiliations:** 1 Kasetsart Agricultural and Agro-Industrial Product Improvement Institute (KAPI), Kasetsart University, Bangkok 10900, Thailand; 2 Faculty of Pharmacy, Chiang Mai University, Chiang Mai 50200, Thailand; 3 Department of Medical Technology, Faculty of Associated Medical Sciences, Chiang Mai University, Chiang Mai 50200, Thailand; 4 School of Agriculture and Natural Resources, University of Phayao, Phayao 56000, Thailand; 5 Food Technology Program, Kanchanaburi Campus, Mahidol University, Kanchanaburi 71150, Thailand

**Keywords:** antioxidant, anti-tyrosinase activity, β-glucan, enzymatic recovery, *Ganoderma lucidum*, mushroom, skin anti-ageing

## Abstract

Mushrooms are incredibly valuable macro fungi that are an important and integral part of the ecosystem. In addition to being used as cuisine, mushrooms have been used for medicinal purposes for many centuries. This research applied a process for recovering β-glucan (BG) from the antler-type fruiting body of *Ganoderma lucidum* as well as tested the biological activities related to cosmeceutical applications. The characterization of complex structure was performed by fourier-transform infrared (FTIR) and nuclear magnetic resonance (MNR) spectroscopies. The obtained extract contained 40.57% BG and 7.47% protein, with the detectable bioactivities of anti-tyrosinase and antioxidation. Consequently, it showed the activity that can be used to whiten the skin by reducing or inhibiting the process of skin pigmentation. The BG also showed moderate activities of anti-collagenase, anti-elastase, and anti-hyaluronidase. The test by the HET-CAM confirmed no skin irritation of the complex extract. Based on human peripheral blood mononuclear cell (PBMC) test, the BG had no significant inhibiting effect on cell viability. In addition, the obtained BG had functional properties higher than commercially available BG, especially oil-binding capacity. These findings provided new insights into the potential application of *G. lucidum* BG as a polymeric material in the cosmeceutical industries.

## 1. Introduction

One of the most prevalent types of polysaccharides is β-glucan (BG), which is constituted by heterogeneous groups of glucose polymers linked together by β-1,3/-1,6-glycosidic linkages. In the food industry, BG is typically used to prepare soups, sauces, beverages, and other food products where it serves as an emulsifier, stabilizing agent, and thickening agent [1]. The hydrophilic property, high absorption capacity, and biocompatibility of BG make it potentially useful in a variety of fields, including skin care [2], drug delivery [3], and wound healing [4].

BG is important for cell function and occurs in significant amounts in biomass. It is abundantly detected in the cell walls of grains, yeasts, bacteria, fungi, and algae. The ongoing research in this field enables us to find the most efficient methods of obtaining glucanic molecules with a variety of biological properties. In order to extract the functional BG from plant and microbial sources, a variety of recovery processes have been used [5,6].

Complex biochemical processes play a role in the aging process of organisms [7]. The main factors that cause aging are free radicals and reactive oxygen species (ROS). They have a crucial role in the aging of the skin [8]. For many years, the pharmaceutical and cosmetic industries have been exploring for substances with protective activity. 

We previously reported the recovery of BG and its functionality derived from a newly identified yeast, an industrial-thermotolerant yeast strain of *Kluyveromyces marxianus* TISTR 5925 [9]. As a by-product of the fermentation of alcohol, the spent yeast cells are recovered and can be used as a source of BG. The study carried out previously revealed that the antioxidant activity of BG was also detected [10], other than its bioactive properties [11]. The presence of numerous valuable bioactive compounds in BG has increased interest in their potential applications. The BG sources that contain a variety of properties are waiting for research, and applications are also still attracting interest.

It has been reported that medicinal mushrooms play a vital role in a variety of disorders because of their numerous metabolic components and nutritional values. *Ganoderma lucidum* belongs to a large group of polypore fungi and has been used as a traditional therapeutic mushroom for over 4000 years [12]. It is a large, kidney-shaped, dark-red mushroom with a glossy surface and a wood-like texture. Conversely, rarely found and known as a young deer horn mushroom, the fruiting body of *G. lucidum* with an antler-like shape is a variation kind of *G. lucidum* [13]. It has been widely known that the cell walls of mushrooms contain a high number of polysaccharides, including BG. Most of mushroom bioactive BG have been found to be complex with peptides, like peptidoglycans, which are not water-soluble and correspond with various bioactivities [14]. Extraction techniques are important for the structure and properties of BG. Various techniques have been applied such as hot water extraction, solvent solution, ultrasound, microwave, or enzyme. To obtain the crude extract, hot water is the most popular BG extraction technique, in which it fractionates only water-soluble BG into the aqueous extract. The water-insoluble BG thus have been isolated by other extraction techniques [15].

The use of enzyme-assisted extraction has several advantages, such as enhanced selectivity, low degradability of the extracted biomolecules, higher extraction yield, and higher quality of extract, especially green extraction because of water and enzyme ap-plications that are of natural origin [16]. Nevertheless, enzyme cost is considered to be one of the most significant factors in comparing the final product price with high purity and quality. To the best of our knowledge, no study has been carried out on the enzymatic recovery and protective activity of the BG of the antler-type fruiting body of *G. lucidum*, despite the fact that there have been numerous studies on the BG of yeast. Studies on its BG are still required. Therefore, we report herein an enzymatic recovery of BG accumulated in the potential sources of antler-type fruiting bodies of *G. lucidum*. The resulting BG samples were characterized for its structure using FTIR and NMR spectroscopies. The functionality of BG, including, swelling capacity, water binding capacity, water holding capacity and oil holding capacity was studied. Furthermore, the antioxidant, anti-tyrosinase, and anti-ageing activities of mushroom BG recovered from antler-type fruiting body of *G. lucidum* were elucidated for its use as a potential polymeric material in cosmeceutical applications.

## 2. Materials and Methods

### 2.1. Mushroom Pretreatment

Freshly harvested antler-type fruiting body of *Ganoderma lucidum*, young deer horn mushroom samples were obtained from the farm at Kasetsart Agricultural and Agro-Industrial Product Improvement Institute, Bangkok, Thailand. Mushroom samples were cleaned, cut, and ground into powder. The resulting powder (particle size 63–150 μm) was obtained by testing sieves (Nonaka Rikaki, Chiyoda, Japan). The mushroom powder was vacuum dried in a vacuum oven (Vac Oven 65.1L 120, Thermo Fisher Scientific, Waltham, MA, USA) at 50 °C until the constant weight was obtained and the moisture content was less than 10%. All powder samples were then airtight packaged and stored at −20 °C before use.

### 2.2. Enzymatic Recovery of BG from Mushroom Fruiting Body

The enzymatic recovery of BG from antler-type fruiting bodies of *G. lucidum* was performed according to some modifications in Vaithanomsat et al. [9]. Water was added (1:15 *w*/*v*), the reaction was stirred with heating to 90 °C for 3 h and cooled down to room temperature. After that, ethanol solution was added (1:15 *w*/*v*) and the reaction was incubated at 4 °C for 15 h. The precipitate was harvested by centrifugation and immediately frozen in liquid nitrogen. Protease enzyme (2.4 AU/mg; Alcalase^®^ 2.4 L FG, Novozymes A/S, Kalundborg, Denmark) was applied at concentration of 1.0% (*w*/*v*). The reaction was incubated at 55 °C for 2 h, subjected to centrifugation, and the precipitates were washed with water. Ethanol (precipitate:ethanol) at a ratio of 1:4 was added. The mixture was centrifuged to obtain the BG precipitate, which then was dried at 40 °C for 48 h under vacuum. *G. lucidum* BG was ground into powder using a multi-purpose grinder (BL-T70-PR2, Toshiba, Tokyo, Japan), subjected to a stainless-steel sieve mesh No. 40, and kept in aluminum foil bags at 4 °C until use.

### 2.3. Determination of BG Content of Antler-Type Fruiting Body G. lucidum

The BG content in *G. lucidum* was analyzed using a Megazyme Mushroom and Yeast Beta-Glucan Assay Kit (Catalog Number K-YBGL, Megazyme International Ireland Ltd., Bray, Ireland) according to the manufacturer’s instruction. Briefly, the 90 mg of dry mushroom sample was mixed with 2 mL of ice cold 12 M H_2_SO_4_. The mixture was vigorously stirred and placed on ice for 2 h. Then, 10 mL of water was added. The reaction was heated to 100 °C for 2 h. The 8 M NaOH solution (6 mL) was added. The volume was adjusted to 100 mL with 200 mM sodium acetate buffer (pH 4.5). After that, the 0.1 mL aliquot was reacted with 0.1 mL of a mixture of exo-1,3-β-glucanase (20 U/mL) plus β-glucosidase (4 U/mL) in 200 mM sodium acetate buffer (pH 4.5) at 40 °C for 60 min. Then, 3.0 mL of GOPOD reagent (glucose oxidase plus peroxidase and 4-aminoantipyrine) was added. The reaction mixture was incubated at 40 °C for 20 min and the absorbance at 510 nm was measured against the reagent blank. The reagent blank contained 0.2 mL sodium acetate buffer (200 mM, pH 4.5) and 3.0 mL GOPOD Reagent. The reference (1 → 3, 1 → 6) BG and all used reagents were available in the Assay Kit.

### 2.4. Analysis of BG Composition

The contents of moisture, protein, lipid, ash, fiber, and carbohydrate in *G. lucidum* BG were analyzed according to the Association of Official Agricultural Chemists (AOAC) [17].

### 2.5. Fourier Transform Infrared (FTIR) Spectroscopy

The FTIR spectrum of *G. lucidum BG* was analyzed on an FTIR spectrometer (Thermo Scientific Nicolet IR200, Waltham, MA, USA) by a total of 128 scans accumulated in attenuated total reflection (ATR) mode with a resolution of 4 cm^−1^. The spectrum was obtained in the range of 4000 to 400 cm^−1^.

### 2.6. Nuclear Magnetic Resonance Spectroscopy (NMR)

The solid-state ^13^C NMR measurements were carried out using a Jeol JNM-ECZ-400R/S1 spectrophotometer (JEOL Ltd., Tokyo, Japan) resonating at 400 MHz, in which spectra were obtained at room temperature, averaging over 5000–33,000 scans. The chemical shifts were referenced to the TMS using adamantane as an external standard.

### 2.7. Analysis of Antioxidant Properties

The antler-type fruiting body *G. lucidum* BG was evaluated for its antioxidant properties following three methods with ascorbic acid as control reference. The *G. lucidum* BG was analyzed using the methodologies by DPPH, FRAP, and ABTS. Concerning the 0.1 g of BG extract, 10 mL of distilled water were added, and the reaction was heated under pressure for 15 min at 121 °C. After that, it was subjected to centrifugation for 10 min at 6000 rpm. The resulting supernatant was tested for antioxidant properties.

#### 2.7.1. 1,1-Diphenyl-2-picrylhydrazyl Radical Scavenging (DPPH) Assay

The 0.1 mM DPPH (1 mL) in ethanol was mixed with 10 mg/mL *G. lucidum* BG (1 mL). The reaction was placed for 30 min in the dark, and then its absorbance at 517 nm was measured using a UV-visible spectrophotometer (Shimadzu UV-160A, Kyoto, Japan). The assay was done in triplicate. The absorbance was calculated to obtain the radical scavenging activity percentage as follows:

% DPPH radical scavenging activity = [(A0 − A1)/A0] × 100

where A0 is the control sample absorbance and A1 is the test sample absorbance.

#### 2.7.2. Ferric Reducing Antioxidant Power (FRAP) Assay

The preparation of FRAP reagent was done by mixing 300 mM acetate buffer (10 μL) with 20 μM FeCl_3_·6H_2_O (1.0 mL), 40 μM 2,4,6-tris(2-pyridyl)-s-triazine in HCl (1.0 mL), and water (1.2 mL). The 10 mg/mL *G. lucidum* BG (60 μL) in 95% ethanol were added, then water (180 μL) and FRAP reagent (1.8 mL). The reaction mixture was placed for 4 min at 37 °C, and the absorbance was then measured at 595 nm using a UV-visible spectrophotometer. The antioxidant properties were evaluated by the ability of the extract to provide free electrons (as a reducing agent). This property was shown by its absorbance in comparison with that of ferrous sulfate (FeSO_4_·7H_2_O) as the standard ranging from 0.10 to 1.00 mmol/L and reported as μmol of Fe(II)/g test sample. The test was assayed in triplicate.

#### 2.7.3. 2,2-Azinobis (3-ethylbenzothiazoline-6-sulphonic Acid) (ABTS) Assay

ABTS assay was used to evaluate the ABTS radical scavenging activity of sample [18]. The calculation of ABTS scavenging activity followed the equation:

% ABTS scavenging activity = [1 − (A/B)] × 100

where A is the UV absorbance of the sample solution containing mixture and B is the UV absorbance of the sample solution-free mixture. IC_50_ were calculated from the graph plotted inhibition percentage against the sample solution concentration using the Graphpad/Prism program version 2.01 (Graphpad Software Inc., La Jolla, CA, USA). Ascorbic acid was used as a positive control. The test was assayed in triplicate.

### 2.8. Analysis of Anti-Tyrosinase Properties

The tyrosinase-inhibiting activity of *G. lucidum* BG was evaluated by adapting the methods of Kubo et al. [19] and Saewan et al. [20]. The sample was prepared at varied concentrations. The 2.5 mM L-DOPA (1 mL) was mixed with 0.1 M sodium phosphate buffer (pH 6.8) (1.8 mL), and the reaction was incubated for 10 min. Then, the test substance (0.1 mL) and tyrosinase (138 U) (0.1 mL) were added, and the reaction was further incubated for 10 min. The reaction was detected by measuring dopachrome via its absorbance at 475 nm using a spectrophotometer. The obtained values were used in evaluation of tyrosinase inhibition percentage following the equation:

% tyrosinase inhibition = [A − (B − C)]/A × 100

where A is the control sample absorbance (no test substance), B is the test sample absorbance with tyrosinase, and C is the test sample absorbance without tyrosinase.

### 2.9. Analysis of Anti-Hyaluronidase Properties

The analysis of the hyaluronidase-inhibiting activity of antler-type fruiting body *G. lucidum* BG was done following the methods of Thring et al. [21]. Briefly, 1.5 U/mL hyaluronidase was mixed with the sample solution and incubated for 10 min at 37 °C, pH 5.3. Then, hyaluronic acid (0.03% *w*/*v*) was added and further incubated for 45 min at 37 °C. After that, acid bovine serum albumin solution, containing acetic acid, sodium acetate, and bovine serum albumin, was added. After 10 min of incubation, the absorbance at 600 nm was measured, and used in determining the percent hyaluronidase inhibition using the following equation:

% hyaluronidase inhibition = [(A − B)/A] × 100

where A is the absorbance of the reaction without sample and B is the absorbance of the reaction with sample. The calculation of IC_50_ was done from the dose–response curve plotted between the % hyaluronidase inhibition versus its log concentrations using the GraphPad/Prism program version 2.01 (GraphPad Software Inc., La Jolla, CA, USA). Oleanolic acid was used as positive control. The test was assayed in triplicate.

### 2.10. Analysis of Anti-Collagenase Properties

The analysis of the collagenase-inhibiting activity of antler-type fruiting body *G. lucidum* BG was done by following Thring et al. [21]. Briefly, 0.5 U/mL collagenase was added in the sample solution and incubated for 15 min at 37 °C, pH 7.5. Thereafter, 2.0 M FALGPA was added, and the absorbance was immediately measured 340 nm in a kinetic mode for 15 min using a multimode detector (SPECTROstar Nano, BMG Labtech, Offenburg, Germany). The resulting values were applied for the determination of the collagenase inhibition percentage following the equation:

% collagenase inhibition = [(A − B)/A] × 100

where A is the absorbance of the reaction without sample and B is the absorbance of the reaction with sample. The calculation of IC_50_ was done from the dose–response curve plotted between the % collagenase inhibition versus its log concentrations using the GraphPad/Prism program version 2.01 (GraphPad Software Inc., La Jolla, CA, USA). Epigallocatechin gallate (EGCG) was used as positive control. The test was assayed in triplicate.

### 2.11. Analysis of Anti-Elastase Properties

The elastase inhibitory activity of antler-type fruiting body *G. lucidum* BG was evaluated based on the method by Thring et al. [18]. Briefly, 7.5 U/mL elastase was mixed with the sample solution and incubated at 25 °C, pH 8 for 20 min. Thereafter, 0.8 mM N-Succinyl-Ala-Ala-Ala-*p*-nitroanilide (AAAPVN) elastase substrate was added. The absorbance of 410 nm was immediately measured in a kinetic mode for 20 min using a multimode detector (SPECTROstar Nano, BMG Labtech, Offenburg, Germany). The resulting values were applied for the determination of the percent elastase inhibition following the equation:

% elastase inhibition = [(A − B)/A] × 100

where A is the absorbance of the reaction without sample and B is the absorbance of the reaction with sample. The calculation of IC_50_ was done from the dose–response curve plotted between the % elastase inhibition versus its log concentrations using the GraphPad/Prism program version 2.01 (GraphPad Software Inc., La Jolla, CA, USA). EGCG was used as positive control. The test was assayed in triplicate.

### 2.12. In Vitro Cytotoxicity Test by MTT Assay

The MTT assay has been regarded as the standard of cytotoxicity assays as it is highly sensitive [22]. Peripheral blood mononuclear cells (PBMCs) were separated from 5 healthy volunteers following the Ficoll–Paque density gradient centrifugation protocol using Lymphoprep™ solution (Axis-Shield, Oslo, Norway). After that, they were cultured in RPMI-1640 medium supplemented with 10% (*v*/*v*) fetal bovine serum (FBS), 1 mM L-glutamine, 100 U/mL penicillin and 0.1 mg/mL streptomycin (Invitrogen^TM^ Life, Carlsbad, CA, USA) under pH 7.2−7.4, 5% CO_2_ atmosphere and 80% humidity (37 °C) for 24 h. The PBMCs (1 × 10^6^ cells/mL) were exposed to the *G. lucidum* BG at varied concentrations (0, 3.125, 6.25, 12.5, 25, 50 and 100 µg/mL) in a 96-well microtiter plate. Following a 48-h incubation, cytotoxic activity was determined using MTT assay. Briefly, 15 µL of 5 mg/mL MTT dye solution (Sigma-Aldrich, Saint Louis, MO, USA) was added into each well and the cells were further incubated for an additional 4 h before the addition of dimethyl sulfoxide (DMSO: 200 µL). Then, the absorbance of formazan was measured at 578 and 630 nm. The % cell viability was calculated following the equation:

% Cell viability = (A/B) × 100

where A is the tested sample absorbance and B is the control absorbance. The test was assayed in triplicate.

### 2.13. In Vitro Irritation Test by Hen’s Egg Test Chorioallantoic Membrane (HET-CAM) Assay

The irritation test of antler-type fruiting body *G. lucidum* BG was performed following Somwongin et al. [23]. The adverse occurrences, including hemorrhage, vascular lysis, and vascular coagulation, were detected after the application of sample solution on the Chorio-Allantoic-Membran (CAM) of the hen’s egg for 5 and 60 min. The irritation index score (IS) was calculated using the following equation:

$$\mathrm{IS}\;=\frac{\left(301-t\left(h\right)\right)}{300}\times 5+\frac{\left(301-t\left(l\right)\right)}{\;300}\times 7+\frac{\left(301-t\left(c\right)\right)\;}{300}\times 9$$

when the initial vascular hemorrhage occurred at time t(h), the initial vascular lysis occurred at time t(l), and the initial coagulation occurred at time t(c). IS was classified as follows: 0.0–0.9 indicated no irritation, 1.0–4.9 indicated mild irritation, 5.0–8.9 indicated moderate irritation, and 9.0–21.0 indicated severe irritation. NSS (0.9% *w*/*v* NaCl) was used as a negative control, whereas 1% (*w*/*v*) sodium lauryl sulfate aqueous solution was used as a positive control. The experiments were carried out 3 times.

### 2.14. Functionality of BG

#### 2.14.1. Water Holding Capacity (WHC) 

WHC was evaluated according to Sangeethapriya and Siddhuraju [24] with modifications. Briefly, 0.02% sodium azide solution (1 mL) was added to 0.5 g of *G. lucidum* BG. The reaction was carefully stirred and placed for 60 min at room temperature. After centrifugation for 15 min at 3000× *g*, the supernatant was carefully discarded, and the residue was weighed (m). WHC was expressed as the amount of water per gram of *G. lucidum* BG (g/g) as follow:

$$\mathrm{WHC}\;(g/g)\;\frac{m\;-\;0.5}{0.5}$$

#### 2.14.2. Water Binding Capacity (WBC) 

WBC was evaluated according to Daou and Zhang [25]. Briefly, 0.02% sodium azide solution (15 mL) was added to 0.5 g of *G. lucidum* BG. The reaction was stirred and placed for 18 h at room temperature. After that, the sample was centrifuged at 3000× *g* for 20 min. The supernatant was carefully discarded, the residue was weighed, and sample was dried under vacuum at 60 °C for 2 h. The dried weight was used for calculation as follow:

$$\mathrm{WBC}\;\left(g/g\right)=\frac{\mathrm{residue}\;\mathrm{weight}\;\mathrm{after}\;\mathrm{centrifugation}\;\mathrm{residue}\;\mathrm{dry}\;\mathrm{weight}}{\mathrm{resideu}\;\mathrm{dry}\;\mathrm{weight}}$$

#### 2.14.3. Swelling Capacity (SC) 

SC was evaluated according to Daou and Zhang [25]. A 0.02% sodium azide solution (10 mL) was added to 0.2 g of *G. lucidum* BG. The reaction was stirred and placed for 18 h at room temperature. Then, the bed volume was recorded. The SC was calculated as follow:

$$\;\mathrm{SC}\;(\mathrm{mL}/g)=\frac{V1\;-\;V0}{W0}$$

where V1 is the volume of the hydrated BG, V0 is the volume of BG prior to hydration, and W0 is the weight of BG prior to hydration.

#### 2.14.4. Oil Holding Capacity (OHC) 

OHC was evaluated according to Sangeethapriya and Siddhuraju [24]. Briefly, refined sunflower oil (10 mL) was added to 0.5 g of *G. lucidum* BG. The reaction was stirred carefully and placed for 60 min at room temperature. After 15 min of centrifugation at 3000× *g*, the supernatant was carefully discarded, and the residue was weighed (m). OHC was expressed as the amount of oil per gram of *G. lucidum* BG (g/g). The OHC was calculated as follows:

$$\mathrm{OHC}\;(g/g)=\frac{m\;-\;0.5}{0.5}$$

### 2.15. Statistical Analysis

All experiment data were evaluated from at least 3 replicates and expressed as mean ± SD. Analysis of variance (ANOVA) was performed by Duncan’s multiple-range test (DMRT) using the SPSS software (SPSS for Windows, SPSS Inc., Chicago, IL, USA) at *p* ≤ 0.05. 

## 3. Results and Discussion

### 3.1. Composition of Antler-Type G. lucidum BG Extract

The composition of the antler-type fruiting bodies of *G. lucidum* extract, and commercial BG is demonstrated in Table 1. The *G. lucidum* extract mainly contained carbohydrates and BG, whereas the contents of fat, protein, and ash were relatively low. In a previous study by Ogbe and Obeka [26] on wild *G. lucidum*, the results showed a similar tendency that the fruiting bodies contained protein (16.79 ± 0.13%), carbohydrate (63.27 ± 0.2%), fiber (7.77 ± 0.34%) and fat (1.52 ± 0.09%). This study, *G. lucidum* extract showed a mean composition of fiber of 30.63%, which is higher than the previous report. The total carbohydrate (carbohydrate and fiber contents) accounts for more than 80% of the proximate composition, which is in accordance with an earlier report of 75.5% [27].

In mushrooms, polysaccharides are present as the cell wall structural components, composing of two polysaccharides major types; known as a rigid cellulose fibrillar and a matrix-like glycoprotein, α- or β-BG [28]. *G. lucidum* polysaccharides have been found to typically contain the pure polysaccharides, and some of them are bound to proteins or peptides. These polysaccharides, including BG, play an important role, vitally influencing the bioactivities [29,30].

A study on BG recovery from kidney-shaped *G. lucidum* using supercritical CO_2_ and pressurized hot water found that at relatively extreme conditions of 155 °C, pH 3.88, and 8.5 MPa of pressure yielded 58.0 ± 2.6% BG, but no mention of its application was made [31]. The high-pressure steaming and enzymatic pre-treatment for *G. lucidum* BG extraction demonstrated that the optimal conditions (15.51 min for high-pressure steaming at 121 °C; 15 lb psi) and 4.16 h for enzymatic hydrolysis) provided 8.05 ± 0.16% BG with anticancer activity [32]. The results obtained in this study revealed high BG content. This might be due to the fact that the polysaccharides contain a number of −OH groups which can be recovered easily by the action of hydrolytic enzyme in the aqueous system containing water. Furthermore, the cell wall of mushrooms is composed mainly of glucans, chitin, and glycoproteins. The commercial enzyme used in this study is a serine endopeptidase, which stereoselectively hydrolyzes ester bonds [33]. Therefore, it is assumed that the protease enzyme might decompose the impurity proteins in the *G. lucidum* cell wall and could increase the extraction yield of BG.

From the results compared to commercially available BG (Table 1), it is noteworthy that the antler-type *G. lucidum* BG obtained by enzymatic recovery contains a relatively high amount of BG and may equivalently have potential health benefits. Then, BG recovered from *G. lucidum* was further characterized and studied for its cosmeceutical applications.

### 3.2. Fourier Transform Infrared (FTIR) Spectroscopy of Antler-Type G. lucidum BG

**Figure 1:**
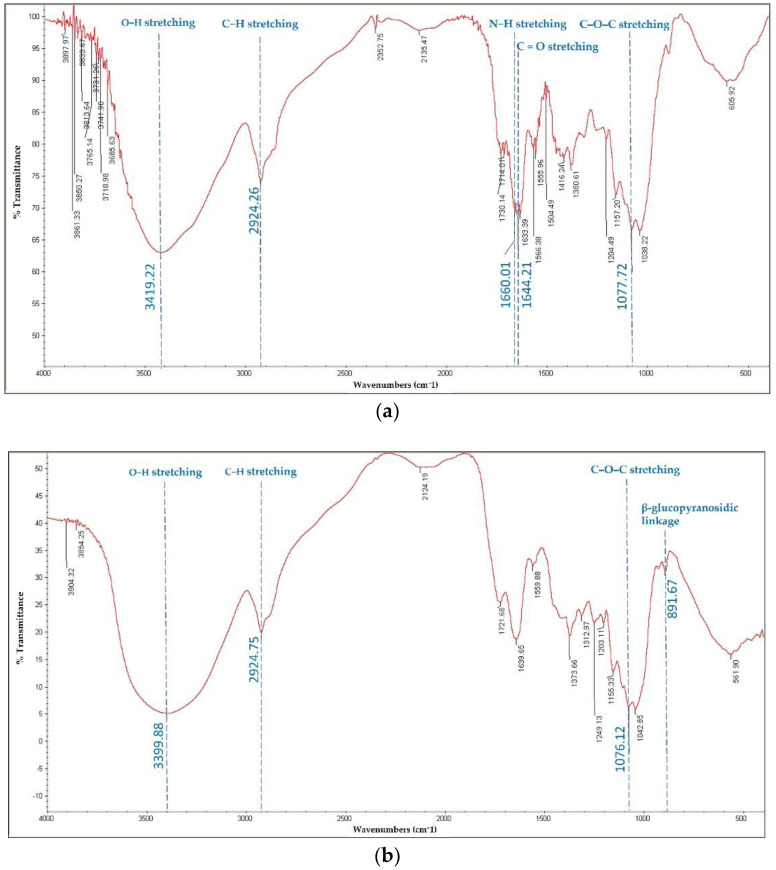
FTIR spectra of commercial BG (**a**); and antler-type *G. lucidum* BG (**b**).

The FTIR spectra of commercial BG and recovered *G. lucidum* BG consisted of the hydroxyl group as strong transmittance bands at 3400 cm^−1^ (strong and wide O–H stretch) and 2924 cm^−1^ corresponds to C-H stretch. The broad band, ascribed to polysaccharides at 1200–800 cm^−1^ corresponded to C–O, C–C stretching, and COH bending [34]. Two weak bands were detected in the commercial BG at 1644 cm^−1^ and 1660 cm^−1^ (Figure 1a), which are assigned to the stretching of C=O and N–H of typical α-chitin amide I bands as an impurity [35]. An observed band at 1076 cm^−1^ is assigned to C–O–C asymmetric stretching vibration of glucan [36]. The specific absorption at 891.67 cm^−1^ revealed the presence of β-glucopyranosidic linkage in *G. lucidum* BG (Figure 1b), which confirmed the results of BG content in the extract (Table 1). Apparently, the spectra of the obtained BG revealed similar absorption peaks in specific bands with commercially available BG.

### 3.3. Nuclear Magnetic Resonance Spectroscopy (NMR) of Antler-Type G. lucidum BG

**Figure 2:**
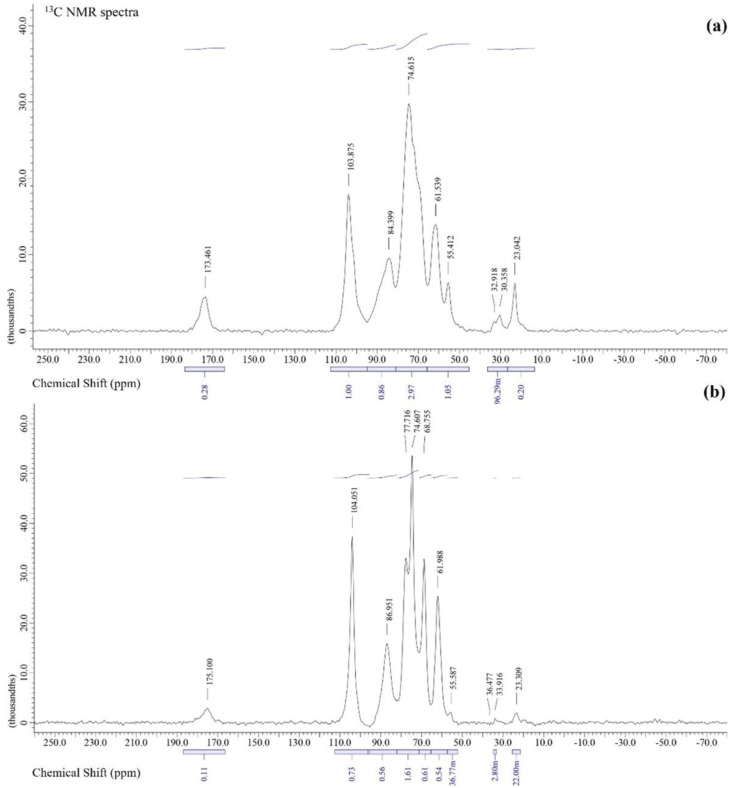
^13^C solid-state NMR spectra of commercial BG (**a**); and antler-type *G. lucidum* BG (**b**).

The ^13^C solid-state NMR spectra of *G. lucidum* BG are depicted in Figure 2. Figure 2a shows the spectrum of *G. lucidum* BG, containing six specific resonance peaks of a linear (1 → 3)-β-D-glucan [37]. The six resonance peaks at 61.9, 68.7, 74.6, 77.7, 86.9 and 104.5 ppm correspond to C6, C4, C2, C5, C3, and C1, respectively. While the resonance peaks at 86.9 ppm correspond to (1 → 3)-linked residues of C3 and show the formation of the β-D-glucan chains in triple helices, the resonance peak at 104.5 ppm is related to the C1 carbon in the β-glycosidic bond. [38]. C4, C2, and C5 signals occur at the chemical shift of 68.7, 74.6 and 77.7 ppm, respectively. Whereas the signals of (1 → 6)-linked C3 and C6 carbons are typically detected. Apart from the signals corresponding to the carbons of (1 → 3, 1 → 6)-β-D-glucan, the significant peaks close to 175 ppm (C=O), 56 ppm (C2), and 23 ppm (CH_3_) occur, which might specify the presence of chitin [39,40], implying the occurrence of impurities in the commercial BG (Figure 2a). Nevertheless, it should be noted that the chemical shifts for the C atoms of this sample have similar values to NMR spectra of commercially available BG (Figure 1b) and BG obtained from *Kluyveromyces marxianus* [9].

Likewise, it is comparable to well-studied BG [11,41,42]. Based on the above results and information from the literature [11,37,41,42], it was suggested that the main polysaccharide component recovered from the antler-type fruiting bodies of *G. lucidum* by an enzymatic process was attributed as BG. In this study, obtaining BG by an enzymatic process was a more effective approach for recovering BG with a considerable purity. 

### 3.4. Protective Activity

Antioxidant activities of antler-type fruiting body *G. lucidum* BG were evaluated by methods of radical scavenging activity on DPPH^•^ and ABTS^•+^ and reported as the *G. lucidum* BG concentration which inhibited the activity of DPPH and ABTS by 50% (IC_50_), respectively. Moreover, the reducing ability (ferric reducing antioxidant power through the reduction of ferric iron (Fe^3+^) to ferrous iron (Fe^2+^) (FRAP), of *G. lucidum* BG was investigated and reported as IC_50_. As shown in Table 2, the *G. lucidum* BG exhibited potent radical scavenging activity against ABTS^•+^, but low ferric reducing antioxidant power. In fact, BG contains a heterogeneous group of glucose polymers, each of which contains a hydroxyl group that can react with other compounds, and they are electron and hydrogen donors. The common structure of BG comprises a main chain of β-glucopyranosyl units, along with side chains with various branches and lengths [43]. Hydroxyl radicals have the strongest reactivity and oxidation power among reactive oxygen species (ROS). The ability to scavenge ROS from the hydroxyl groups of BG is considered a precious property for the prevention of various diseases and aging. In this study, it was shown that BG recovered from the antler-type mushroom by the enzyme method exerts significant antioxidant activity by radical scavenging ability to transfer one electron to the free radical of ferric reducing power (FRAP), ABTS and DPPH in the assays. The radical scavenging activity of BG was very similar to that of ascorbic acid, a common chemical agent and widely used an antioxidant. Antioxidants would offer some protection against premature aging because the human body, particularly the skin, is regularly exposed to stressful environmental factors including UV radiation and pollutants, which generate an enormous number of aggressive oxidants that damages all biological skin cell membranes [44].

Apart from the antioxidant activities, antler-type fruiting body *G. lucidum* BG also possessed a potent anti-tyrosinase effect, as shown in Table 3, particularly when the substrate of the enzyme was L-tyrosine. The anti-tyrosinase effect of *G. lucidum* BG was almost comparable to that of kojic acid, a well-known whitening agent widely used in the cosmetic industry [45]. As a result, it might appropriately whiten the skin by decreasing or blocking the pigmentation process.

The extracellular matrices in skin tightening and resilience, including collagen, hyaluronan, and elastin, are known to be degraded by the collagenase, hyaluronidase, and elastase enzymes. Thus, the degradation of collagen and elastin causes wrinkles and an aging appearance of the skin [46]. As a result, inhibiting these enzymes would retard the aging of the skin. The anti-aging activities of antler-type fruiting body *G. lucidum* BG were investigated by means of the inhibitions of collagenase, hyaluronidase, and elastase, which are shown in Table 4. The results show slight inhibition on collagenase, hyaluronidase, and elastase activities when compared with those of standards. Skin fibrous protein degradation is primarily mediated by metalloproteinases, a superfamily of protease enzymes whose catalytic action involves a metal, which includes collagenase, elastase, and tyrosinase [47]. Collagenase and elastase are zinc-containing metalloproteinases. The hydroxyl group of BG may bind to the Zn^2+^ ion within the enzyme molecules, thus preventing it from binding with the substrate. On the other hand, tyrosinase contains Cu^2+^, which can be bound to the hydroxyl group of BG. Katsube et al. [48] also reported that the purified polysaccharide showed inhibitory activity on hyaluronidase, and the inhibition of hyaluronidase was the result of substrate competition.

The obtained results suggested that antler-type fruiting body *G. lucidum* BG had protective effect including antioxidant, anti-tyrosinase, and mild anti-ageing activities and could be the active ingredient in cosmeceuticals for the skin pigmentation retardation. The aforementioned findings showed that antler-type *G. lucidum* BG can also be used as an alternative to chemical agents for protective activity in cosmetic applications.

In order to confirm the safety of antler-type fruiting body *G. lucidum* BG for further cosmeceutical application, the irritation and toxicity tests were performed in vitro through the HET-CAM assay and MTT assay, respectively. The irritation assay result is shown in Table 5. This result demonstrates that the 1% *w*/*v* sodium lauryl sulfate (positive control) with an irritation index score (IS) of 15.07 ± 0.08 showed severe irritation induction, whereas 0.9% *w*/*v* sodium chloride (negative control) and *G. lucidum* BG induced no sign of irritation on CAM with IS of 0.00.

**Figure 3:**
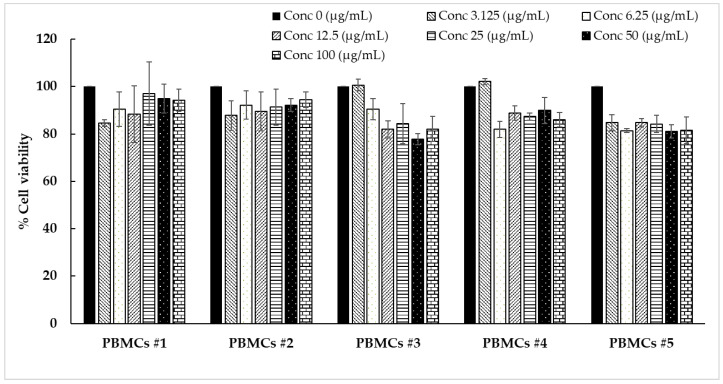
Effect of antler-type *G. lucidum* BG at various concentrations on cell viability of peripheral blood mononuclear cell (PBMC) from 5 healthy volunteers (#1–#5). Data were expressed as Mean ± SD values from 3 replications.

Figure 3 shows the effect of antler-type *G. lucidum* BG at various concentrations on % cell viability of peripheral blood mononuclear cells (PBMCs). The result shows the safety profile of *G. lucidum* BG on PBMCs with high % cell viability and IC_50_ value more than 100 µg/mL. As a result, the *G. lucidum* BG was considered as safe for further application in the products. 

### 3.5. In Vitro Functional Properties of Antler-Type G. lucidum BG

In addition to its protective activity, the functionality of antler-type *G. lucidum* BG was also evaluated for its use in cosmetic applications. Structurally, polysaccharide structures contain several hydroxyl groups which are capable of binding with water molecules. In the application of cosmeceutical, polysaccharides are extensively applied in various cosmetic dosage forms as a natural thickening agent [49]. They are also widely applied to hydrate facial and body skin as a natural moisturizing agent. Glycosyl units in BG typically contain three hydroxyl groups. In polymer chains of BG, each hydroxyl group has the capability to form hydrogen bond through one or more molecules of water. Therefore, in aqueous systems, BG molecules are able to take up, hold water, swell, and subsequently provide more hydration to the skin [50].

Table 6 presents the in vitro functional properties, including water-holding capacity (WHC), water-binding capacity (WBC), swelling capacity (SC), and oil-holding capacity (OHC) of antler-type *G. lucidum* BG and commercial BG.

As presented in Table 6, there were no significant differences in WHC, WBC and SC between the obtained BG and commercial BG. These observed values in this study demonstrated that BG exhibited hydrophilic behavior. Interestingly, *G. lucidum* BG showed relatively high OHC when compared to commercial BG. High values of OHC could relate to the high capability of BG to act as an emulsifier in aqueous formulations containing oil. 

The extraction and fractionation methods of *G. lucidum* polysaccharide varied significantly due to the purposes of each study. From the previous studies of kidney-shaped *G. lucidum*, it has been noted that the extreme ultrasonic-assisted and microwave-assisted processes might destroy molecules of polysaccharide by vibration, orbit, and destruction during extraction [49,51]. The different types of studied mushrooms might have a significant impact on the difference in BG content. The properties that subsequently alter the biological activities of the obtained BG may also be affected by differences in some extraction conditions, such as extraction temperature, time, and the power of equipment. The hydroxyl radical scavenging activity of BG was also increased with an increase in molecular size, as earlier reported by Kofuji et al. [10]. Overall, the obtained BG using enzymatic recovery would be taken into consideration as a multi-functional polymeric material for anti-aging, moisturizing, and whitening purposes in cosmetic application.

## 4. Conclusions

In conclusion, we have demonstrated an effective method for the recovery of the antler-type fruiting body *Ganoderma lucidum* BG using an enzymatic method, which could exhibit antioxidant, anti-tyrosinase, and anti-ageing activities, suggesting that it can be used to whiten the skin by decreasing or blocking the pigmentation process. Anti-collagenase, anti-elastase and anti-hyaluronidase activities were also detected at moderate levels in the extract. The HET-CAM test confirmed that the *G. lucidum* BG did not cause skin irritation. In a human PBMC, it was found that the BG extract had no significant inhibitory effect on cell viability. Our results demonstrated that young deer horn mushroom, *G. lucidum* is considered as a potential source of biologically active substances having medicinal values. The findings open up a new possibility to apply the antler-type fruiting body *G. lucidum* BG as a polymeric material in the cosmetic industries.

## Figures and Tables

**Table 1:** Composition of commercially available *G. lucidum* BG and antler-type *G. lucidum* BG extract.

BG Sources	BG (%*w*/*w*)	Carbohydrate (%*w*/*w*)	Fiber (%*w*/*w*)	Protein (%*w*/*w*)	Fat (%*w*/*w*)	Moisture (%*w*/*w*)	Ash (%*w*/*w*)
*G. lucidum*	48.69 ± 0.63 ^a^	54.61 ^b^	30.63 ± 0.22 ^a^	7.47 ± 0.10 ^b^	0.12 ± 0.04 ^b^	6.79 ± 0.28 ^a^	0.38 ± 0.13 ^a^
Commercial BG	40.57 ± 0.90 ^b^	59.29 ^a^	20.76 ± 0.02 ^b^	11.61 ± 0.12 ^a^	2.61 ± 0.01 ^a^	5.30 ± 0.01 ^b^	0.43 ± 0.21 ^a^

^a,b^ Means ± SD in the column with different small superscript letters a and b indicate significant difference at *p* < 0.05 level (*n* = 3).

**Table 2:** Antioxidant activities of antler-type *G. lucidum* BG.

Antioxidant Activity	IC_50_ (mg/mL)	
	*G. lucidum* BG	L-Ascorbic Acid
DPPH^•^ inhibition	18.34 ± 5.77 * (R^2^ = 0.9584)	0.05 ± 0.00 (R^2^ = 0.9584)
ABTS^•+^ inhibition	0.07 ± 0.00 (R^2^ = 0.9947)	0.06 ± 0.00 (R^2^ = 1.0000)
Ferric reducing power	18.38 ± 1.68 * (R^2^ = 0.9584)	0.03 ± 0.00 (R^2^ = 0.9985)

IC_50_ = the concentration of *G. lucidum* BG inhibiting the activity of DPPH, ABTS and Ferric reducing power by 50%. Asterisk (*) denotes significant differences in means between *G. lucidum* BG and L-ascorbic acid determined using *t*-test (*p* < 0.001).

**Table 3:** Anti-tyrosinase activity of antler-type *G. lucidum* BG.

Anti-Tyrosinase Activity	Inhibition (%)	
	*G. lucidum* BG	Kojic Acid
Substrate: L-tyrosine Substrate: L-DOPA	97.66 ± 0.59 *	99.11 ± 0.48
	24.13 ± 1.34 **	89.80 ± 0.17

The final concentration of *G. lucidum* BG and kojic acid in each tested system was 0.5 mg/mL. Asterisk (*) denotes significant differences in means between *G. lucidum* BG and kojic acid determined using *t*-test, * *p* <0.05 and ** *p* < 0.001.

**Table 4:** Anti-ageing activity of antler-type *G. lucidum* BG.

Anti-Ageing Activity	Inhibition (%)		
	*G. lucidum* BG	EGCG	Oleanolic Acid
Anti-collagenase	21.03 ± 2.64 *	66.07 ± 1.26	ND
Anti-elastase	26.19 ± 3.37 *	89.61 ± 3.04	ND
Anti-hyaluronidase	29.26 ± 4.48 *	ND	81.35 ± 1.55

The final concentration of *G. lucidum* BG, EGCG, and oleanolic acid in each tested system was 0.5 mg/mL. Asterisk (*) denotes significant differences in means between *G. lucidum* BG and EGCG or oleanolic acid determined using *t*-test (*p* < 0.001). ND, not determined.

**Table 5:** Effect of 1% *w*/*v* sodium lauryl sulfate solution (positive control), 0.9% *w*/*v* sodium chloride solution (negative control), and antler-type *G. lucidum* BG on hen’s egg chorioallantoic membrane before (0 min) and after exposure for 5 and 60 min.

Samples		Before (0 min)	5 min	60 min	Irritation Score
Negative control	0.9% *w*/*v* Sodium chloride	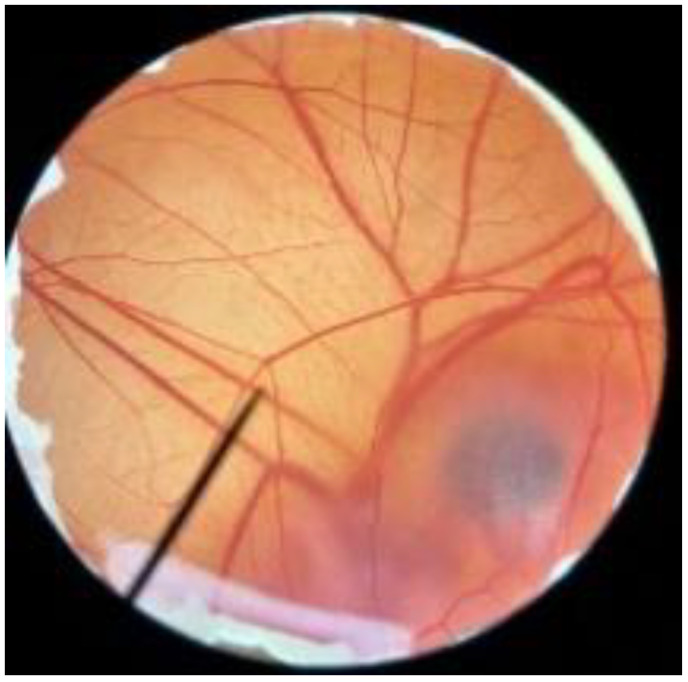	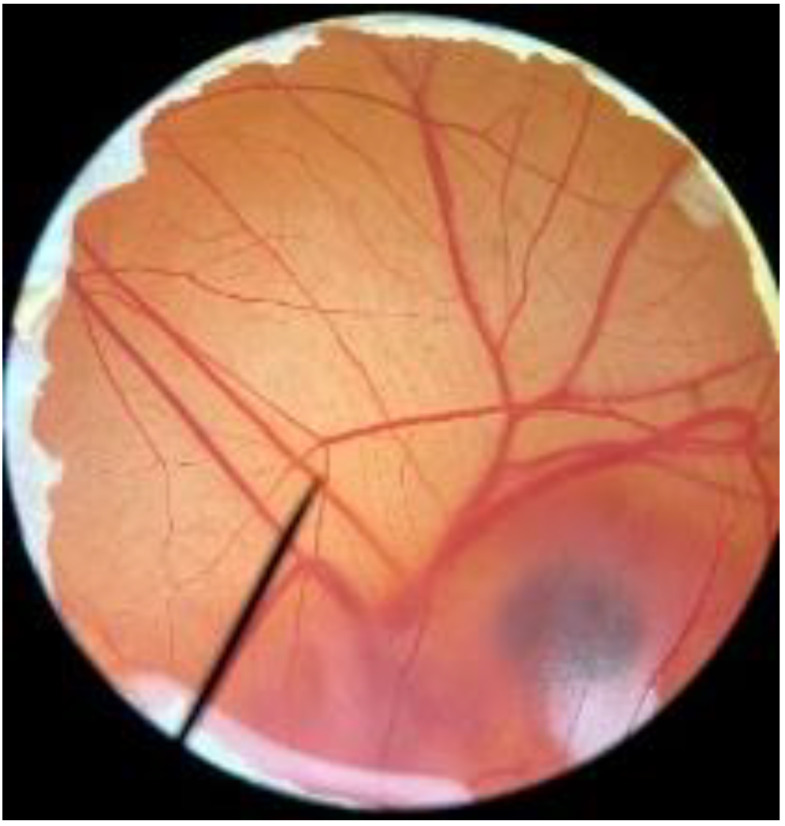	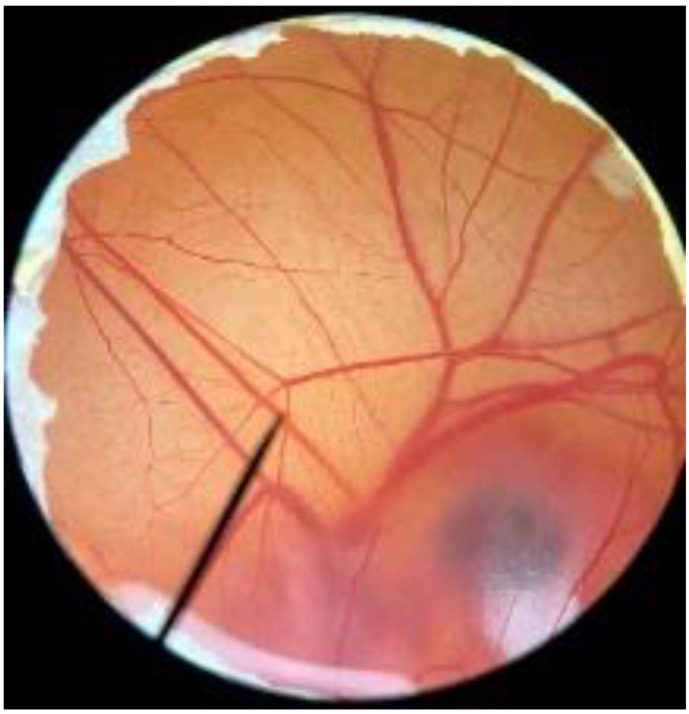	0.00
Positive control	1% *w*/*v* Sodium lauryl sulfate	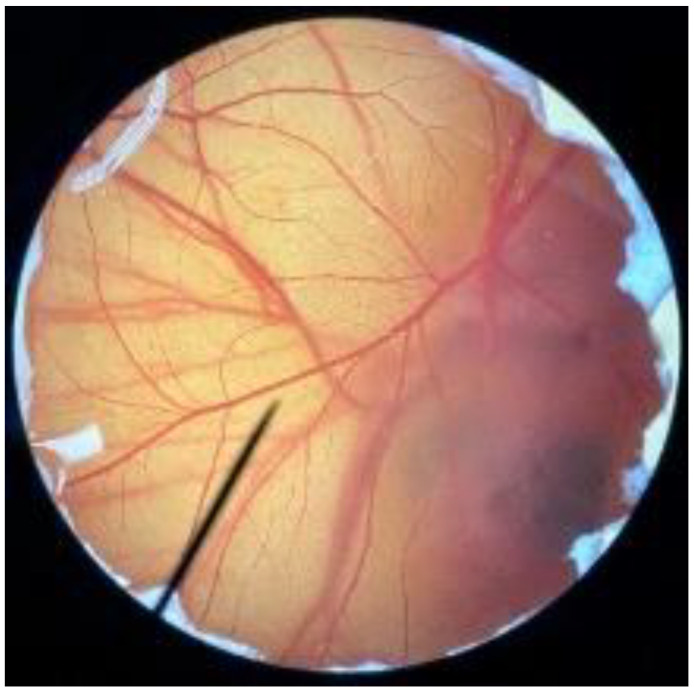	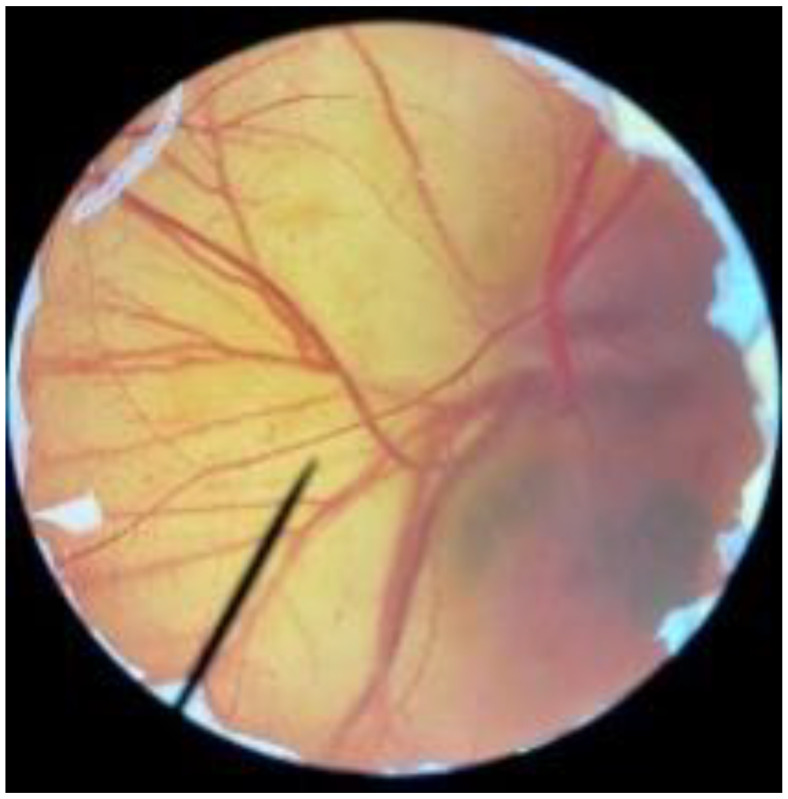	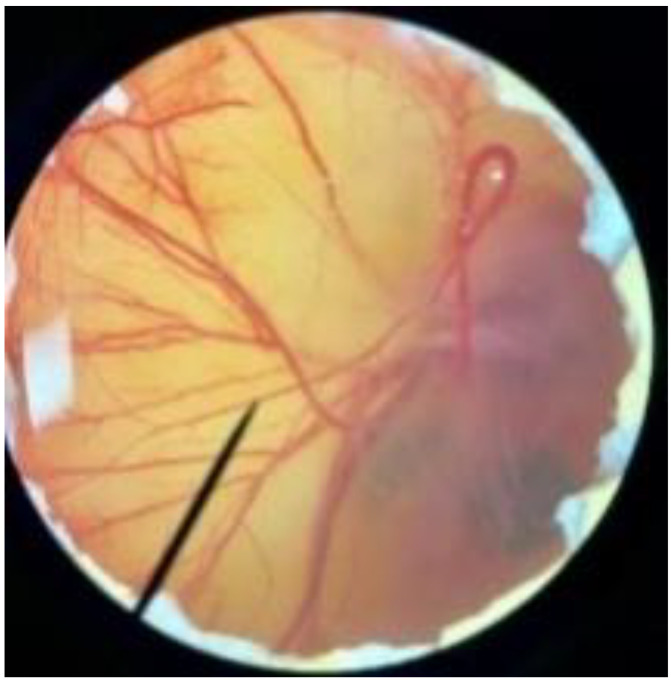	15.07 ± 0.08
Sample	*G. lucidum* BG	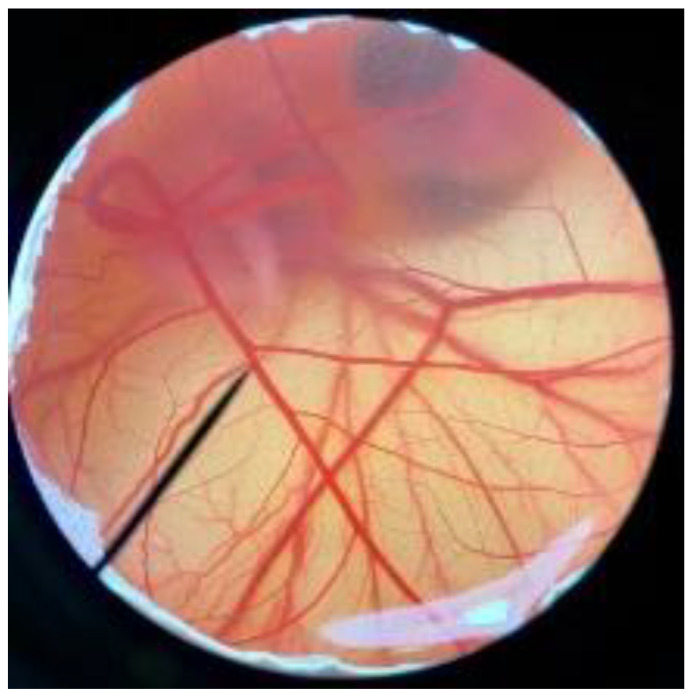	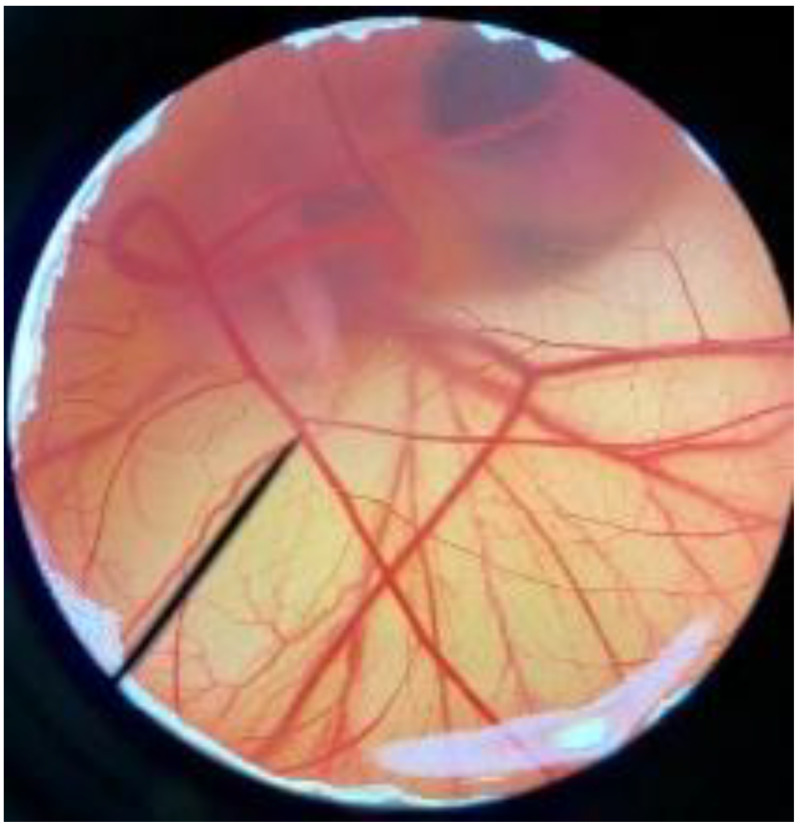	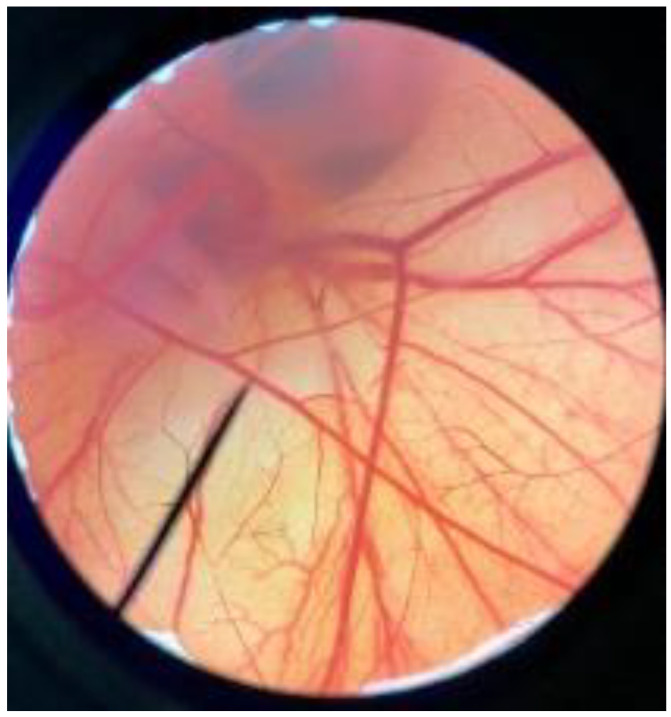	0.00
Vehicle control	DI water	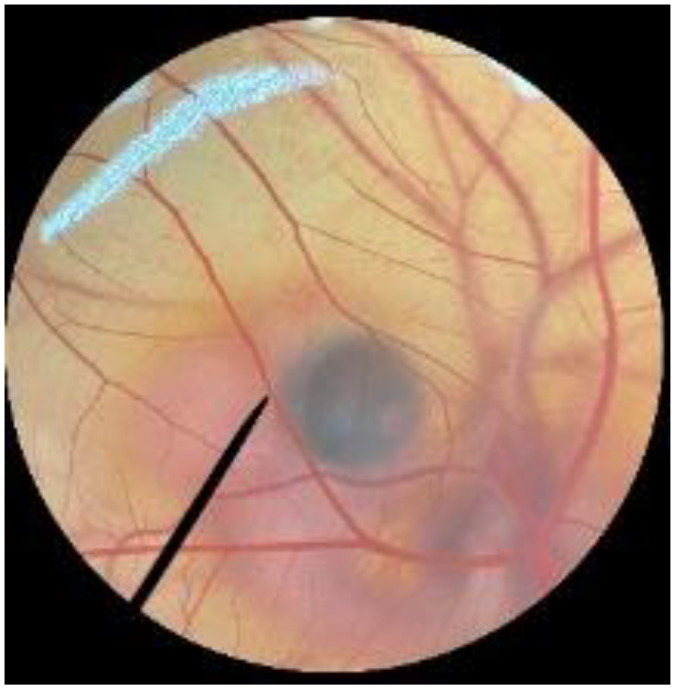	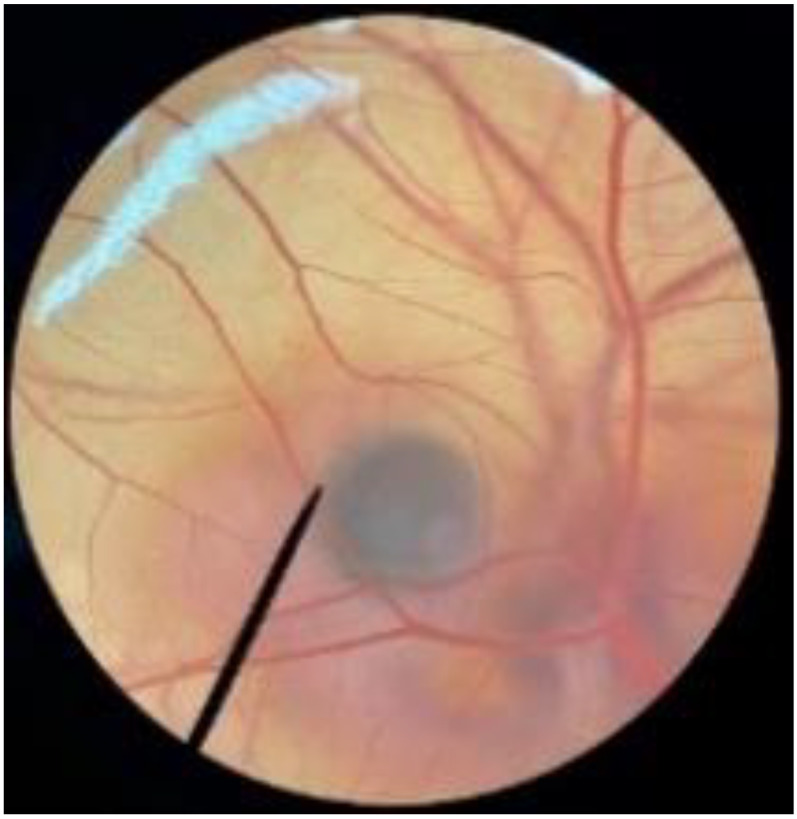	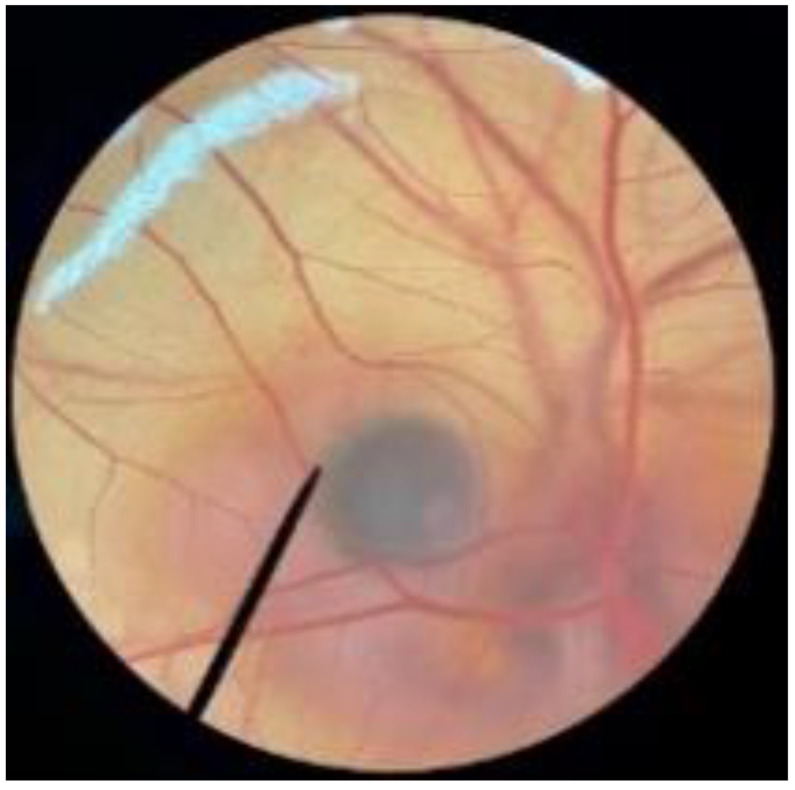	0.00

**Table 6:** Water-holding capacity (WHC), water-binding capacity (WBC), swelling capacity (SC), and oil-holding capacity (OHC) of antler-type *G. lucidum* BG and commercial BG.

BG Sources	WHC (g/g)	WBC (g/g)	SC (mL/g)	OHC (g/g)
*G. lucidum*	1.96 ± 0.01 ^a^	0.17 ± 0.01 ^a^	41.34 ± 0.53 ^a^	8.15 ± 0.04 ^a^
Commercial BG	1.97 ± 0.01 ^a^	0.13 ± 0.01 ^b^	41.79 ± 0.27 ^a^	3.00 ± 0.32 ^b^

^a,b^ Means ± SD in the column with different small superscript letters a, b indicate significant difference at *p* < 0.05 level (*n* = 3).

## Data Availability

Data is contained within the article.
